# Gastroprotective activity of the resin from *Virola oleifera*

**DOI:** 10.1080/13880209.2016.1251467

**Published:** 2016-12-09

**Authors:** Ana Claudia Hertel Pereira, Dominik Lenz, Breno Valentim Nogueira, Rodrigo Scherer, Tadeu Uggere Andrade, Helber Barcellos da Costa, Wanderson Romão, Thiago Melo Costa Pereira, Denise Coutinho Endringer

**Affiliations:** a Pharmaceutical Sciences Graduated Program, University Vila Velha, Vila Velha, Brazil;; b Morphology Department, Federal University of Espírito Santo, Vitória, Brasil;; c Federal Institute of Education, Science and Technology (IFES), Vila Velha, Brazil

**Keywords:** Antioxidant, bicuíba, epicatechin, myristicaceae, peptic ulcer, polyphenols

## Abstract

**Context:** The resin from the trunk wood of *Virola oleifera* (Schott) A. C. Smith (Myristicaceae) is used in folk medicine to hasten wound repair and to treat pain and inflammatory conditions, and our previous report indicated the anti-oxidative properties in other oxidative stress model.

**Objective:** To investigate the protective effects of resin from *V. oleifera* in two experimental models of gastric ulcer oxidative-stress dependent.

**Materials and methods:** Plant material was collected and the resin was subjected to partitioning with organic solvents. The buthanol fraction was subjected to chromatographic and spectrometric methods for isolation and structural elucidation. The resin was quantified for polyphenols and flavonoids by colorimetric methods. Furthermore, the antioxidant activity of resin was determined by three different methods. The ulcers were induced acutely in Swiss male mice with ethanol/HCl and indomethacin using single-doses of 10 and 100 mg/kg. The gastroprotection of the experimental groups was comparable to reference control lansoprazole (3 mg/kg).

**Results:** The high content of polyphenols (∼82%) and the presence of epicatechin and eriodictyol were determined. The LD_50_ was estimated at 2500 mg/kg. At minimum (10 mg/kg) and maximum (100 mg/kg) dosage of resin, both in ethanol/HCl as indomethacin ulcer induction models demonstrate reduction of lesions (minimum: ∼97% and ∼66%; maximum: ∼95% and ∼59%).

**Discussion:** The gastroprotection might be related to tannins, phenolic acids and flavonoids present in the resin by antioxidant properties.

**Conclusions:** The results indicate that this resin has gastroprotective activity probably associated with the presence of phenolic antioxidant substances.

## Introduction

*Virola oleifera* (Schott) A. C. Smith (Myristicaceae), commonly known as ‘bicuíba’, ‘bocuva’, ‘bicuíva’, ‘ucuúba’, ‘candeia-do-caboclo’, is a tree of the Atlantic Forest (Colombo & Joly 2010). The oil extracted from seeds of *Virola* species is popularly used for rheumatic pains, bronchial asthma, tumors in the joints, intestinal worms, halitosis, haemorrhoids and skin diseases (Rodrigues 1980). The bark when grated produces a resin that is used against chronic wounds, diarrhoea, leucorrhoea and haemoptysis (Rodrigues 1980; Bôa et al. 2015). Despite its widespread use in popular medicine against a myriad of diseases, there were no scientific reports in literature supporting the use of crude resin as an anti-ulcer agent.

The phytochemical analysis performed in this study determined the content of phenolic substances, as there are reports about the gastroprotective properties of these substances (Mota et al. 2009; Bansal & Goel 2012), and they have also been identified in several species of *Virola* bark (Hiruma-Lima et al. 2009) among other plants (Thirunavukkarasu et al. 2009; Alimi et al. 2010). Recently, data from our laboratory (Bôa et al. 2015) demonstrated the presence of phenolic acids in the resin from *V. oleifera* and an antioxidant effect *in vitro* and *in vivo*.

The hypothesis is that the resin of *V. oleifera* has gastroprotective activity, due to the presence of flavonoids and phenolic compounds. Therefore, the aim of this study was to characterize the chemical resin of the plant *Virola oleifera*, existing in the Atlantic Forest in the state of Espírito Santo, and evaluate its antioxidant and gastroprotective effect. In the search of new potential analgesic/anti-inflammatory agents with gastroprotective properties, the present study evaluated its effects in experimental models of gastric ulcer.

## Materials and methods

### Animals

Male Swiss mice *Mus musculus* (25–40 g) from the Experimental Monitoring Laboratory of University Vila Velha (UVV) were used in the experiments. The animals were fed a normal chow diet and water *ad libitum* under standard conditions of dark–light cycle of 12 h and temperature (23 ± 3 °C). Prior to all gastroprotective assays the animals were 24 h fasting, with access only to 10% glucose solution *ad libitum*. All biological assays were approved by Ethics, Bioethics and Animal Welfare of UVV (CEUA-UVV 150/2011) and were performed according to the international principles accepted from NIH 85-23.

### Chemicals and reagents

Pyrogallol (≥99%), quercetin (≥98%), butylhydroxytoluene (BHT), 2,2′-azino-bis(3-ethylbenzothiazoline-6-sulfonate) (ABTS) were purchased from Sigma-Aldrich Co. (St. Louis, MO). Potassium persulfate, sodium acetate trihydrated, TPTZ and iron chloride were purchased from VETEC Química Fina LTDA. Lansoprazole were purchased from Henrifarma^®^ (Henrifarma Produtos Químicos e Farmacêuticos LTDA, São Paulo-Brazil). Other reagents were of HPLC grade or analytical grade. Milli-Q plus water (Millipore, Missouri, NH) was used throughout this study, and all solutions were prepared immediately before use.

### Resin material

The resin of *Virola oleifera* (Schott) A. C. Smith (Myristicaceae) was collected in February 2012 from the district of Fazenda Guandu, in Afonso Claudio (Espírito Santo – Brazil, S 20° 13490′ W 041° 06692′). The resin was collected with authorization (IEMA 6 2 9/09) and in accordance with the Brazilian law (Resolution 29, 12/06/2007), which states that no special permission is necessary to collect samples of essential oil or fixed oil, or when the tested material remains similar to the raw material (Provisional Statement 2.186-16, 08/23/2001). The plant material was verified by D.Sc. Luciana Dias Thomaz, Department of Botany, Federal University of Espírito Santo, where the voucher specimen was deposited (VIES 19648). The fluid exudate was obtained from 0.5 cm deep incisions in the tree trunk. The resin was collected in aseptic plastic containers and transferred to an amber glass vial and was kept in +4 °C until the analysis. The fluid exudate was then subjected to drying at 40 °C. After drying, it was grained and 24 g of dried resin was obtained.

### Isolation and identification of substances

An aliquot of the dried resin (10 g) was re-suspended in water and partitioned with dichloromethane (3 × 20 mL), followed by buthan-1-ol (3 × 20 mL). The fractions were concentrated to residue, providing 0.0047 g of dichloromethane fraction (VOD), 1.06 g of buthanol fraction (VOB) and 7.91 g of the aqueous fraction (VOA). The butanol fraction (1.0 g) was re-suspended in methanol and applied on a column of Sephadex^®^LH-20 (19 cm × 2 cm, 6 g, Sigma Aldrich) using HPLC grade methanol as an eluent. Fractions (10 mL) were collected and analyzed by TLC [Si gel plates, ethyl acetate:formic acid:water (18:1:2)] and grouped, resulting in 22 fractions (F1–F22).

The F7, which was tested positive for polyphenols in the presence of ferric chloride (FeCl_3_ 1%), was dried and rechromatographed on silica gel column 60 M (Macherey Nagel 0.04–0.0063 mm; 230–400 mesh) (24 cm ×2.5 cm, 15 g), eluting first with ethyl acetate:formic acid:water (18:1:2) and gradually decreasing the percentage of ethyl acetate until its complete removal (Sun et al. 2007). Fractions (10 mL) were collected and analyzed by TLC [ethyl acetate:formic acid:water (18:1:2)], resulting in a total of 15 fractions (F7-1 to F7-15). The fraction F7-2 and F7-3 that had showed positive result with FeCl_3_, with visualization of a single band, underwent spectrometric analysis (ESI-MS and NMR).

### Identification of substances by ultra-high resolution and accuracy mass spectrometry (ESI(−)FT-ICR MS, ESI(−)MS/MS)

The experiments were performed on an ultra-high resolution and accuracy mass spectrometer (model 9.4 T Solarix, Bruker Daltonics, Bremen, Germany), ESI(−)-FT-ICR MS. The ESI(?)FT-ICR spectrum was acquired in the *m/z* region of 200–2000. Briefly, the sample was dissolved in a methanol/ammonium hydroxide mixture (99.9/0.1 v/v %), thus resulting in a final concentration of 1 μg/mL. The parameters of ESI(−) source were: (a) capillary voltage: + 3000–3500 V; (b) end plate offset = −100 V; (c) temperature and gas flow drying: 180 °C e 4 L/min; and (d) nebulizer gas pressure: 0.5 bar. The ion accumulation time in the hexapole for 0.01 s was followed by transporting to the analyzer cell through the electrostatic lens system. Each spectrum was acquired by accumulating 100 scans of time-doming transient signals in the length of 4 mega-point time-domain data sets. A resolving power (*m*/Δ*m*_50%_ ≈ 500,000, in which Δ*m*_50%_ is the full peak width at half-maximum peak height) of *m/z* 400 and a mass accuracy of <1 ppm provided unambiguous molecular formula assignments for singly charged molecular ions (Colati et al. 2013). Additionally, tandem mass spectrometry experiments (ESI(−)-FT-ICR MS/MS) were also performed for the ions of *m/z* 289, 579 and 869. ESI(−)-FT-ICR MS/MS spectra were acquired with an isolation window of 1.0 (*m/z* units) and 19–27 V of collision energy. All spectra were processed using software package Compass Data Analysis (Bruker Daltonics, Bremen, Germany). The DBE was calculated according to the formula DBE = c – h/2 + n/2 + 1, where c, h, and n are the numbers of carbon, hydrogen, and nitrogen atoms, respectively, in the molecular formula.

### Spectrophotometric quantification of total of polyphenols and flavonoids and tannins

The determination of total polyphenols, tannins and total flavonoids in the resin were performed as described by Krepsky et al. (2012). The quantification of total polyphenols was expressed as a percentage calculated as pyrogallol equivalent and total as quercetin equivalent.

### Determination of antioxidant activity

#### ABTS method

The antioxidant activity of the resin was determined by the method of radical scavenging using ABTS according to Re et al. (1999) with slight modifications. Initially, the radical ABTS^•+ ^was formed by the mixture of 7.0 mM of ABTS (ethanol 50%) with 2.45 mM of potassium persulfate (deionized water). This reagent was kept under +4 °C for at least 16 h. Before use, the reagent was diluted in ethanol 50% until an absorbance of 1.0 (± 0.01) in 734 nm was obtained. In 96-well microplates, 270 μL of ABTS^•+ ^radical and 30 μL of each concentration of the compounds were added. In blank well, 30 μL of ethanol was added. After 6 min of reaction in the dark, the absorbance was determined in a 734 nm using a microplate reader (SpectraMax 190, Molecular Devices, Sunnyvale, CA). The antioxidant activity was expressed in I% = (Abs1 – Abs0) × 100, where Abs0 is the blank absorbance and Abs1 is the absorbance of the test. The antioxidant activity of the resin was compared with the action of the synthetic antioxidant BHT.

#### FRAP method

The antioxidant activity of the resin was also analyzed by the FRAP (Ferric Reducing Antioxidant Power) method according to Benzie and Strain (1996), with modifications. For the preparation of FRAP reagent, 25 mL of sodium acetate trihidrated (0.3 M; pH 3.6) was mixed to 2.5 mL of TPTZ solution (10 mM) in HCl 40 mM and 2.5 mL of iron chloride aqueous solution (20 mM), in a total of 30 mL of FRAP solution, that was used immediately after preparation. An aliquot of 30 μL of test solutions were added to 270 μL of FRAP reagent. For blank, 30 μL of ethanol was mixed with 270 μL of FRAP reagent. After 5 min of reaction, the absorbance was read in 595 nm using an absorbance microplate reader. The antioxidant activity was express in I% = (Abs1 – Abs0) × 100, where Abs0 is the blank absorbance and Abs1 is the absorbance of the test. The antioxidant activity of the resin was compared with the action of the synthetic antioxidant BHT.

### Gastroprotective activity assay

#### HCl/ethanol-induced ulcer

The anti-ulcerogenic activity of the dried resin was studied by ethanol/HCl-induced gastric ulcer. The experiments were performed as described by Oyagi et al. (2010) with adaptations. Mice were divided into six groups of five animals. Each group had fasted prior to receiving an oral dosage of saline (5 mL/kg), lansoprazole (3 mg/kg) (Olate et al. 2012) or dried resin solubilized in saline (at the dosages of 1, 10, 100 mg/kg) and negative control. After 50 min, all groups were treated orally with 0.2 mL of 0.3 μM HCl/60% ethanol solution (ethanol/HCl) for the induction of gastric ulcer, except the negative control group. Animals were euthanized 1 h after the administration of ethanol/HCl, and the stomachs excised and inflated by 1% formalin injection. The extent of the lesion was measured and the lesion index was expressed as the sum of injured areas divided by the area of the stomach as described by Szelenyi and Thiemer (1978). Finally, the data were compared with the data of the negative groups, and the amount of lesions was expressed as relative unities (RU). The stomachs were then stored for histological evaluation.

#### Indomethacin-induced ulcer (40 mg/kg)

The experiment was performed according to the method of Djahanguiri (1969) with modifications. Briefly, gastric lesions were induced with indomethacin (40 mg/kg), dissolved in 1.0 mL of sodium bicarbonate 0.5 mol/L) and administered intraperitoneally to mice after fasting. Solubilized resin (10 and 100 mg/kg), lansoprazole (3 mg/kg) (Olate et al. 2012) and vehicle (saline 5 mL/kg) were administrated orally 30 min before the induction of gastric lesions. Five hours after the induction of ulcers, the animals (*n* = 5) were anesthetized and euthanized. The stomachs were removed and, subsequently, inflated. Gastric damage was determined as described above.

### Acute oral toxicity assay

The experiments with male Swiss mice were performed in accordance with the ethical principles established by the Brazilian College of Animal Experimentation (COBEA, 1991). The animals were between 8 and 12 weeks old at the beginning of the experiments, with a mean weight of 25 ± 2 g.

Five days before starting the protocol, the animals were housed in cages, one per cage, with free access to water and food. Throughout the experiment, the animals had free access to food and water, except for the 3–4 h of fasting they were subjected to prior administration of the resin and 1–2 h after administration. After the fasting period of 3–4 h, time (*t*) = 0, the animals were weighed. The resin was administered as a single dose (2000 mg/kg, as specified by protocol OECD 423) by gavage using a suitable intubation cannula (gavage).

Three animals were used for control group, and three for the test group. The control group received the vehicle (drinking water) orally, in an amount equal to the average volume (0.2 mL) of the compound, both in a single dose. After treatment, all animals were closely observed for 4 h and then daily for 14 days; any behavioural changes that occurred were noted.

Additionally, the weight of each animal was monitored during the experiments and at the end of the 3–4 h fasting period as well as along the 14 days after the administration of the studied dose. After the 14th day, any deaths that occurred were recorded to calculate the LD50, and all of the surviving animals were anesthetized with sodium thiopental 120 mg/kg intraperitoneally, then thoracotomy was performed. Blood was collected by cardiac puncture in the right ventricle and it was added to a tube without the addition of an anticoagulant.

The samples were used for biochemical tests of liver function markers (albumin, aspartate aminotransferase [AST], alanine aminotransferase [ALT], alkaline phosphatase [ALP], and γ-glutamyl transferase [GGT]) and renal function markers (creatinine, urea and total protein). The animals were then euthanized and their kidneys and livers were extracted and weighted.

After all of the reported procedures were completed, the animals were frozen and all medical waste was collected according to the required protocol. The results observed in the experiments were recorded for each animal, showing the number of animals used, the number of animals presenting signs of toxicity, the number of animals found dead during the test and the number of animals that were euthanized after 14 days. The description and the time course of the toxic effects and the reversibility and the necropsy findings were also documented.

### Biochemical analyses

Biochemical analyses were performed using a semi-automatic biochemical analyzer (LabmaxPlenno-Labtest, Minas Gerais, Brazil). The biochemical parameters measured were urea (Labtest^®^ cod. 104.2, Minas Gerais, Brazil, linearity ≥300, sensitivity of 0.051–0.061 mg/dL, colorimetric method, 600 nm), creatinine K (Labtest^®^ cod.96.2, linearity of 0.2–12 mg/dL, sensitivity of 0.583–0.648 mg/dL, colorimetric method, 510 nm), total protein (Labtest^®^ cod.99.1, linearity ≥14 g/dL, sensitivity of 0.017–0.019 g/dL, colorimetric method, 345 nm), albumin (Labtest^®^ cod.19.1, linearity ≥6 g/dL, sensitivity of 0.0091–0.0099 g/dL, colorimetric method, 630 nm), aspartate aminotransferase (AST, Labtest^®^ cod.109.2, linearity ≥400 U/L, sensitivity of 01.430–1.943 U/L, cinetic UV-IFCC method, 340 nm), γ-glutamyltransferase (GGT, Labtest^®^ cod.105.2, linearity ≥700 U/L, modified Szasz method, 405 nm), alanine-aminotransferase (ALT, cod.108.2, linearity ≥400 U/L, sensitivity of 01.469–2.029 U/L, cinetic UV-IFCC method, 340 nm) and alkaline phosphatase (ALP, Labtest^®^ cod.79.4, linearity ≥1500 U/L, colorimetric assay, 405 nm).

### Histological analysis

The stomachs of animals undergoing gastric ulcers were opened from the greater curvature and preserved in a solution of 10% buffered formalin. They were then processed for subsequent embedment. The resulting tissue samples blocks were cut into sections of 2.5 μm. Two slides were prepared from each sample containing three consecutive cuts, one of the slides was stained with hematoxylin–eosin (HE) and the other with HE and periodic acid-Schiff (PAS) for staining structures containing a high proportion of mucins in stomach. Samples were analyzed with an OlympusAX70 microscope system with a digital camera (ZeissAxioCamERc5Smodel, Oberkochen, Germany).

### Image analysis

The images were obtained with the aid of Semiprofessional Digital Camera Canon Power Shot SX10IS model in resolution 12 megapixel photographs by way of the short distances (macro mode), without flash and positioned 20 cm from the blade to be photographed. The tissue was placed between two glass sheets and pressed lightly to allow the observation of the entire area inside of the stomach. The images were then analyzed using the free software CellProfiler^®^, Carpenter Lab (Cambridge, MA).

### Statistical analysis

The results of the chemical evaluation were expressed as mean ± standard deviation, while the results of the biological evaluation were expressed as mean ± SEM. Statistical comparisons between the different groups were performed by one-way analysis of variance (ANOVA) followed by Tukey’s *post hoc* test.

The results of the biochemical analyses and animal’s body weight for the acute toxicity assay were expressed as mean ± standard deviation (SD). Data were submitted to analysis of variance (ANOVA); the homoscedasticity was evaluated using the Bartlett test and the significance of the difference between the means was determined by *post-hoc* test using the Tukey method, adjusted for multiple comparisons, both with a significance accepted at *p* < 0.05. Statistical analyses were performed using GraphPad software (GraphPad Software Inc., San Diego, CA).

## Results

*Virola oleifera* was well known by the population in Afonso Cláudio (Baliano et al. 2015; Bôa et al. 2015) and this study showed the antiulcer effect of resin of *V. oleifera* using two models of antiulcer activity. For the resin collected from *V. oleifera*, the total polyphenolic content was 82.34 g equivalent pyrogallol/100 g of resin and tannins was 67.66 g/100 g of resin. The total flavonoid content was 48.257 ± 28.27 mg equivalent quercetin/100 g of resin.

**Figure 1.**
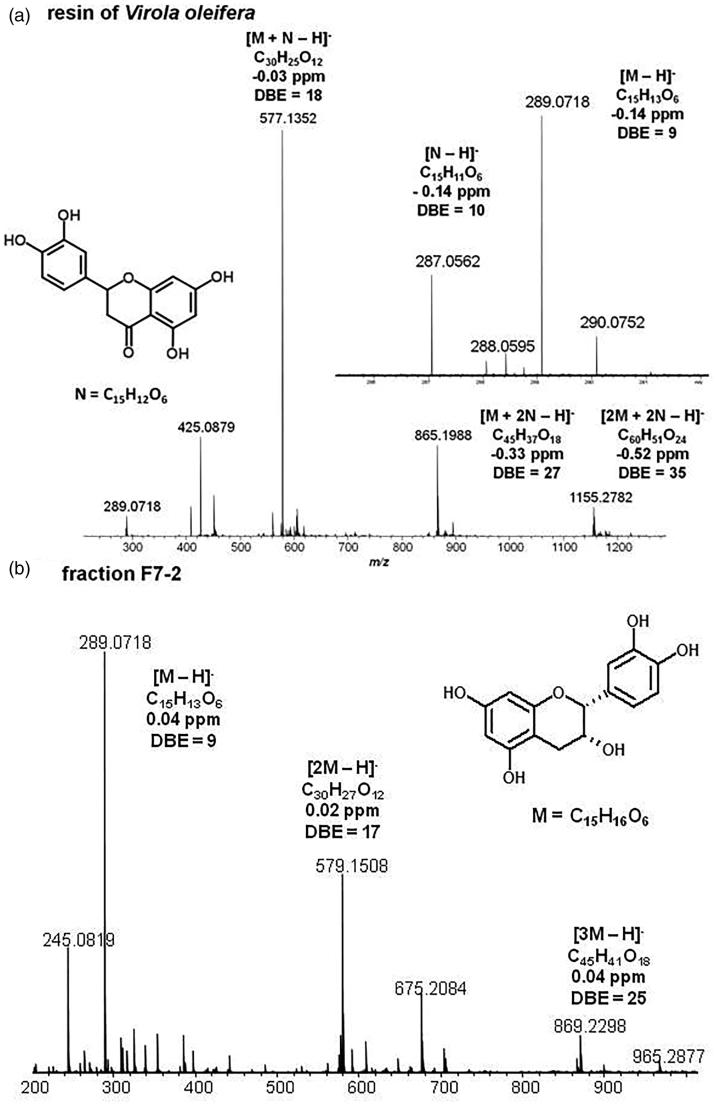
ESI(−)-FT-ICR mass spectra of resin of *V. oleifera* and fraction F7-2.

The resin of *V. oleifera* (Figure 1(a)) and the product isolated (Figure 1(b)) were analyzed by ESI-FT-ICR MS. The isolated product was identified as epicatechin (Figure 1(b)), where the presence of signals of *m/z* 289.0718, 579.1508 and 869.2298 corresponding to [M − H]^−^, [2M − H]^−^ and [3M − H]^−^ ions, respectively, where M = C_15_H_16_O_6_ and DBE =9 (Figure 1(b)).

**Figure 2.**
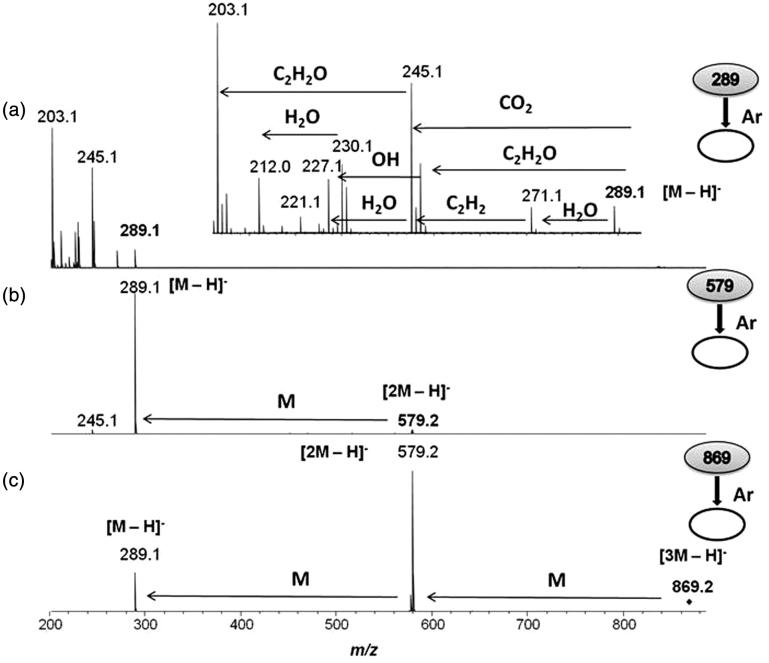
ESI(−)-MS/MS for ions of (a) *m/z* 289, (b) *m/z* 579 e (c) *m/z* 869.

The structure and the connectivity of the epicatechin molecule was confirmed from the ESI(−)-MS/MS spectra (Figure 2(a–c)) for the ions of *m/z* 298, 579 and 869, respectively. The epicatechin was also detected, in lower proportion, in the resin of *V. oleifera* (Figure 2(a)), as [M − H]^−^ ion of *m/z* 289.0718 and as [M + N − H]^−^, [M + 2N − H]^−^ and [2M + 2N − H]^−^ ions of *m/z* 577.1352, 865.1988 and 1155.2782, respectively, where N = C_15_H_12_O_6_ and DBE =10 correspond to a 3,4,5,7-tetrahydroxyflavanone known as eriodictyol, being your structure proposed in the inset of Figure 2(a).

Results are in good agreement with that reported by Pezet et al. (2001). All three compounds were also confirmed by ^1^H and ^13^C NMR (Lobô et al. 2008). The resin of *V. oleifera* showed a high-antioxidant activity when compared to a synthetic antioxidant (BHT), and in agreement of the results found by Bôa et al. (2015).

Total antioxidant capacity of resin of *V. oleifera* determined by spectrophotometric ABTS, FRAP and DPPH methods is depicted in the Table 2. The resin showed a notable antioxidant capacity comparable with the control BHT.

**Table 2.** Total antioxidant capacity of resin of *Virola oleifera* determined by spectrophotometric ABTS, FRAP and DPPH methods.

	Resin of *V. oleifera*		BHT	
Method	IC_50_ (μg/mL)	R^2^	IC_50_ (μg/mL)	*R*^2^
ABTS	7.33 ± 0.09	0.9889	7.48 ± 0.17	0.9590
FRAP	7.54 ± 0.07	0.9939	6.98 ± 0.08	0.9964
DPPH	7.42 ± 0.03	0.9985	4.40 ± 0.01	0.9900

Values represented by mean ± standard deviation of the mean.

Focusing in phenolic substances, all the substances found in the resin and the isolated epicatechin, possess gastroprotective action (Mota et al. 2009) and was found in other species of *Virola* (Hiruma-Lima et al. 2009). As for the acute toxicity assay of the resin, two animals died after receiving the resin (2000 mg/kg), but the other animals did not show any macroscopic changes. This fact indicated that the resin of *V. oleifera* would be classified in category 5, with an estimated median lethal dose (LD_50_) of 2500 mg/kg according to protocol of test 423 (OECD 2001).

No significant change in body weight or correlation to liver/body weight or kidney/body weight was noticed among the other animals,. The biochemical analysis showed no difference between the groups (Table 1). The groups tested also did not present differences in behaviour or eating habits.

**Table 1.** Results of the biochemical analysis 14 days after the acute dose of the *V. oleifera* resin.

Biochemical analysis	Control	Resin 2000 mg/kg	*p* Value
Albumin (g/dL)	1.91 ± 0.12	1.81 ± 0.09	0.2571
Alkaline phosphatase (U/L)	89.67 ± 13.16	98.67 ± 21.89	0.3660
ALT (U/L)	47.17 ± 3.331	40.00 ± 5.50	0.2759
AST (U/L)	198.8 ± 49.33	167.7 ± 17.36	0.6827
Creatinine (mg/dL)	0.29 ± 0.02	0.32 ± 0.03	0.2609
GGT (U/L)	1.50 ± 0.34	1.50 ± 0.50	0.5000
Total protein (g/dL)	5.54 ± 0.10	5.700 ± 0.17	0.2144
Urea (mg/dL)	57.83 ± 1.40	51.25 ± 3.27	0.0341

Values represented by mean ± standard error of the mean. *n* = 6.

**Figure 3.**
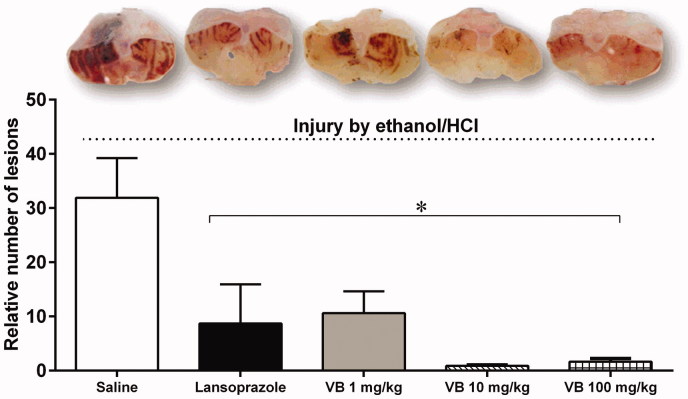
Gastroprotective effect of resin from *V. oleifera* in mice. At the top, macroscopic images of the stomachs of mice subjected to injury by ethanol/HCl. The bar graph shows the mean number of lesions in the respective groups. The values are represented as mean ± S.E.M. Relative number of lesions compared to negative control group. *p < 0.05 vs. control mice; VB: resin of *V. oleifera*. *n* = 5.

In the evaluation of the gastroprotective activity, our study is the first to demonstrate benefits with the resin of *V. oleifera* in two different experimental models, with equal or better efficacy (respectively) when compared to lansoprazole, a classical proton pump inhibitor. Ethanol/HCl induced intense gastric mucosal damage in the control group of mice that received vehicle alone (Figure 3). Oral treatment of 1, 10, 100 mg/kg of *V. oleifera* significantly reduced the gastric mucosal damage and quantitative reduction (VB1 = 10.62 ± 4.01 RU; VB10 = 0.89 ± 0.27 RU; VB100 = 1.65 ± 0.61 RU, *p* < 0.05) compared with the control-treated group (31.89 ± 7.33 RU). The dosages greater than 1 mg/kg showed maximum protective response. The lansoprazole group (3 mg/kg) also demonstrated gastroprotection (8.7 ± 7.22 RU, *p* < 0.05).

**Figure 4.**
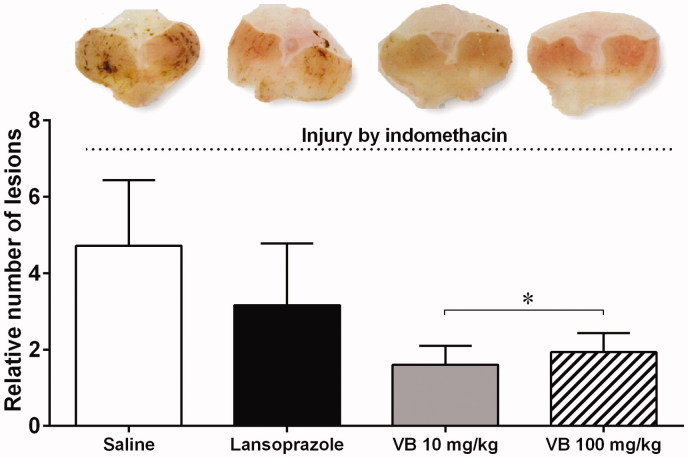
Gastroprotective effect of resin *V. oleifera* in mice. At the top, typical macroscopic images of the stomachs of mice subjected to injury by indomethacin. The bar graph shows the mean number of lesions in the respective groups. The values are represented as mean ± standard error of the mean. Relative number of lesions compared to negative control group. *p < 0.05 vs. control mice. *n* = 5.

The gastroprotective effect of *V. oleifera* on indomethacin-induced gastric damage was macroscopically determined in mice (Figure 4). Macroscopic lesions with evident borderlines in various forms and sizes were dispersed irregularly on all stomach surfaces in the stomach tissue of the control mice that received only indomethacin (4.72 ± 1.71 RU). The mice that received oral treatment of 10 and 100 mg/kg of *V. oleifera* showed same gastric mucosal protection (VB10 = 1.61 ± 1.09* RU; VB100 = 1.93 ± 1.12* RU, *p* < 0.05), while the lansoprazole group did not show acute gastroprotection (3.16 ± 1.62 RU).

**Figure 5.**
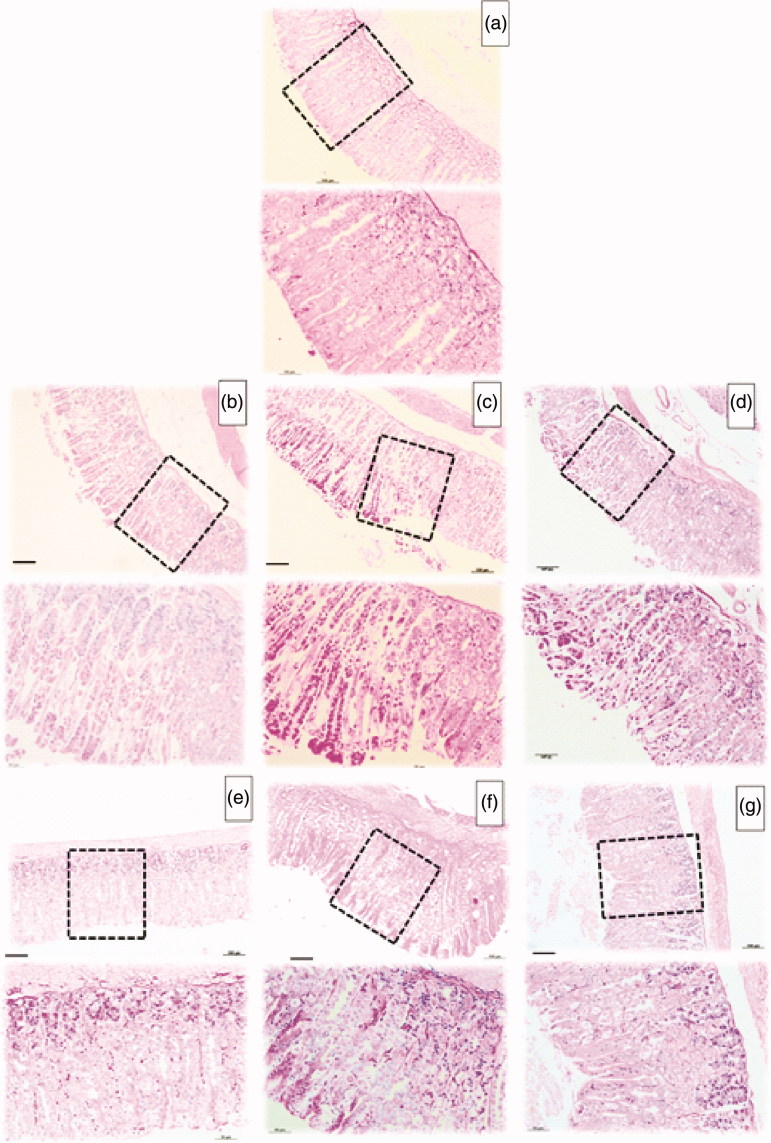
Stomachs photomicrographs stained with hematoxylin and eosin. (a) Histological section of negative control group. (b) Microscopic images of lesions induced by ethanol/HCl in gastric mucosa pretreated with vehicle. (c) Microscopic image of lesions pretreated with lansoprazole 3 mg/kg, (d) Microscopic image of lesions pretreated with *V. oleifera* at 1 mg/kg, (e) at 10 mg/kg (f) 100 mg/kg and (g) 250 mg/kg. Scale: 100 μm (top micrograph) and 50 μm (lower micrograph). *n* = 5.

Additionally, histopathological analysis confirmed that pretreatment with *V. oleifera* prevented ethanol/HCl and indomethacin-induced histological damage in the superficial layers of the gastric mucosa with congestion by HE staining. Compared with the negative control group, ethanol/HCl administration induced a disruption of the superficial region with epithelial cell loss and intense oedema formation (Figures 5(a,b), respectively). In this model, histological analysis showed that *V. oleifera* at the same dosages prevents the development of gastric ulcer (Figures 5(d–g), respectively).

**Figure 6.**
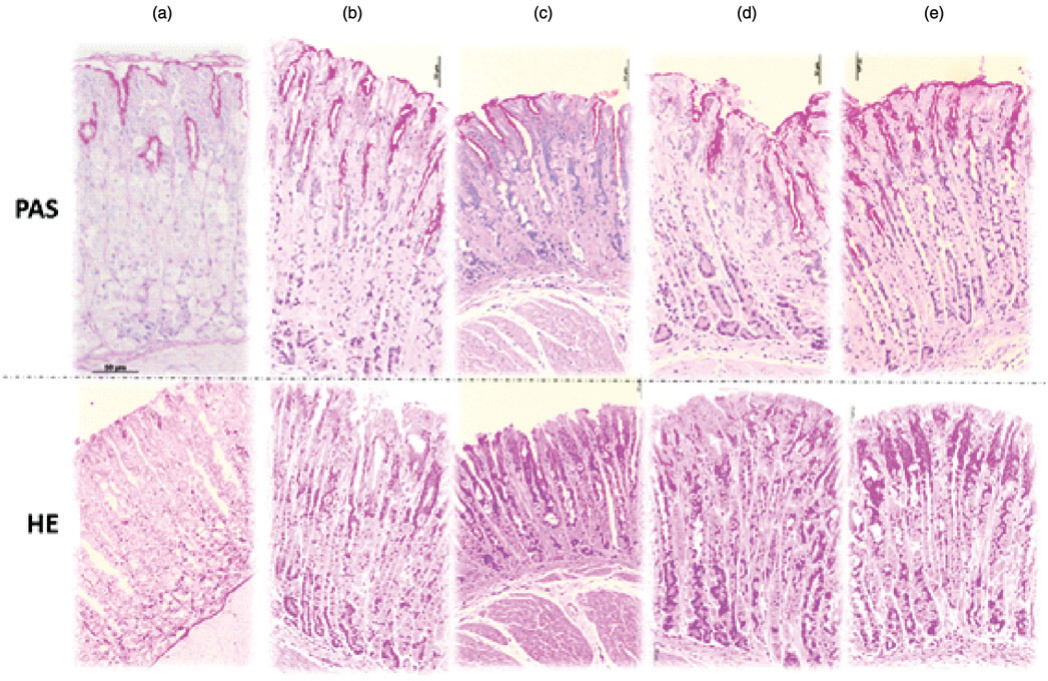
Effect of resin *Virola oleifera* on the histological evaluation of ulcer model induced by indomethacin. Sections stained with periodic acid-Schiff (PAS) and hematoxylin and eosin (HE) (a–e). Sections made in gastric mucosa in negative control group (a). Microscopic appearance of indomethacin-induced lesions in the gastric mucosa pretreated with vehicle (b). Microscopic appearance of indomethacin-induced lesions in the gastric mucosa pretreated with lansoprazole 3 mg/kg (c). Microscopic appearance of lesions pretreated with 10 mg/kg (d), and 100 mg/kg (E) of *V. oleifera*. Scale =50 μm (upper and lower micrograph, respectively). *n* = 5.

As the indomethacin model, compared with the negative control group, indomethacin administration induced a disruption of the superficial region (HE stain) with the loss of mucus analyzed by Periodic Acid Schiff stain (Figures 6(a,b)). Histological analysis showed that both dosages (10 and 100 mg/kg) of *V. oleifera* prevented this damage (Figures 6(d,e)).

## Discussion

Ethanol and HCl quickly infiltrate the gastric mucosa and cause membrane damage, exfoliation of cells, erosion and ulcer formation. Consequently, there is an increase in mucosal permeability together with the release of vasoactive products from leucocytes that can lead to vascular injury, oxidative stress and necrosis (Oyagi et al. 2010). On the other hand, it has been suggested that indomethacin induces gastric damage by inhibiting the release of protective factors like cyclooxygenase-1 (COX-1), prostaglandin E_2_ (PGE_2_), bicarbonate and mucus, besides reducing antioxidant mechanisms while increasing oxidant factors (Halici et al. 2005). Independent of original mechanism, both models could be well-accepted as oxidative stress-induced stomach disease, since the mechanism of ulcer induction might be mediated by reactive oxygen species (ROS) (Oyagi et al. 2010).

According to the chemical analyses and the *in vivo* assays observed, the maintenance of activity by *V. oleifera* in the presence of indomethacin may be attributed to an adaptive cytoprotection independent in part on PG pathway. Then, we proposed three main mechanisms of gastroprotection by *V. oleifera*: (1) the antioxidant effect of tannins, phenolic acids and flavonoids; (2) the mechanical protection mainly by tannin content and (3) other regulatory mechanisms, explained below.

Firstly, the antioxidant effect shown by our resin *in vitro* and recently *in vivo* by Bôa et al. (2015) corroborates with other studies that associate gastroprotection in the presence of phenolic substances and antioxidant effects (Mota et al. 2009; Thirunavukkarasu et al. 2009; Bansal & Goel 2012; Monteforte et al. 2014). We speculate that the polyphenols might protect against peptic ulcer by many pathways such as cytoprotection, re-ephitelialization and suppressing oxidative damage, as mentioned by Farzaei et al. (2015). Among the polyphenols, the flavonoids have been extensively studied in relation to gastroprotection (Mota et al. 2009). Moreover, the eriodictyol, isolated in the resin, seems to decrease the oxidative stress and so gastric lesions (Lee 2011). As another example, the quercetin, flavonoid measured on the resin from *V. oleifera* (Bôa et al. 2015), also seems to reverse damage caused by the imbalance between redox defense system (Coşkun et al. 2004). As discussed by Rios et al. (2002), some procyanidins may limit the oxidative stress, reduce the anti-inflamatory response and by that contributing to decrease the lesion in the mucosa. Moreover, several studies have related the gastroprotective activity of extracts and isolated compounds of catechin or their oligomers to its antioxidant activity (Alimi et al. 2010; Bansal & Goel 2012; Monteforte et al. 2014). Epicatechin, identified in the resin, shows peroxyl radical scavenging activity *in vitro* (Yilmaz & Toledo 2004) contributing directly to the reduction of oxidative stress.

Secondly, the mechanical protection of the resin may be associated with high tannin content, being capable of complexing with proteins or glycoproteins, causing their precipitation over the mucosa, forming an impenetrable layer to harmful agents (Goldstein & Swain 1965; Rios et al. 2002; Thirunavukkarasu et al. 2009). This hypothesis cannot be ruled out, as all treatments and ulcer agent were orally administered. The capacity of complexing of these substances can also result in inactivation of enzymes and preventing the back-diffusion of acid and digestive enzymes (Goldstein & Swain 1965; de Jesus et al. 2012). Other substances present in *V. oleifera* (Bôa et al. 2015) such as gallic acid and ferulic, quercetin and catechin could contribute to mucus preservation, maintaining its integrity (Figure 3).

Additionally, the phenolic compounds are also capable of generating increased formation of new capillaries and fibroblasts (Thirunavukkarasu et al. 2009). The phenolic acids, including ferulic and gallic quantified in *V. oleifera* (Bôa et al. 2015) are beneficial to mucosal protection (Badary et al. 2006; Laine et al. 2008; Barros et al. 2008, Pal et al. 2010). These substances may inhibit the activity of lipoxygenase, decreasing of inflammatory response (Badary et al. 2006), besides stimulating the PGs biosynthesis (Alanko et al. 1999). Also, with the multifactorial aetiology of gastric ulcers, it is estimated that the gastroprotective action of certain phenolic acids, including ferulic, is related not only to a cytoprotective role but also to the H_2_ receptor antagonism (Barros et al. 2008). In the literature it is described that gallic acid and quercetin inhibit the release of histamine, and may have an antisecretory effect (Kahraman et al. 2003; Kim et al. 2006) reducing the damage on the gastric mucosa (Glavin & Szabo 1992).

## Conclusion

In summary, gastroprotective effects associated to the resin of *V. oleifera* could be justified by composition being rich in phenolic compounds. Further studies are needed to be conducted to assess its toxicity and its mechanism of action. Analyses regarding the activity of the various pathways involved in gastric ulcer induction and/or oxidative stress are also suggested.
